# Editorial: Functions and regulation of p90RSK and its family: mechanisms, roles in diseases, and implications in therapeutics

**DOI:** 10.3389/fcell.2025.1648101

**Published:** 2025-07-09

**Authors:** Ling Lin, Nhat-Tu Le, Kebin Hu

**Affiliations:** ^1^ Department of Medicine and Department of Cell and Biological Systems, Penn State University College of Medicine, Hershey, PA, United States; ^2^ Department of Cardiovascular Sciences, Houston Methodist Research Institute, Houston, TX, United States

**Keywords:** p90RSK, signal transduction, kidney diseases, cancers, RSK (ribosomal S6 kinase), transcription

The 90 kDa ribosomal s6 kinases (RSKs) are a group of serine/threonine kinases that were initially found in *Xenopus* to be responsible for phosphorylating ribosomal protein S6 (Erikson and Maller, 1985). The RSK family consists of 4 isoforms (RSK1-4), of which RSK1 is also named as p90RSK. RSKs play important roles in the Ras-mitogen-activated protein kinase (MAPK) signaling cascade and are the direct downstream effectors of extracellular signal-regulated kinase (Erk1/2). Erk1/2 activation directly phosphorylates and activates RSKs (Anjum and Blenis, 2008; Carriere et al., 2008), which, in turn, activate various intracellular signaling events through selection of different phosphorylation substrates to modulate diverse cellular processes (Lin et al., 2019a), such as cell proliferation, survival, and motility, and/or mediate intercellular signaling relays to regulate the phenotypes of other cells. Aberrant activation of RSK has been found in various human diseases including kidney, lung, cardiovascular diseases, and cancers, and plays a critical role in the pathogenesis of organ dysfunction and damage.

The goal of this Research Topic is to provide updated and latest knowledge regarding the functions and signaling mechanisms underlying the regulation of p90RSK and its family members, as well as their roles in disease pathogenesis and the potential therapeutics targeting these important kinases. Four quality manuscripts were included in this specific Research Topic. Three of these publications are original research articles with emphasis on p90RSK-mediated mechanisms in cancers. Ramos JW group delineated RSK isoform-specific transcriptional gene regulation by comparing transcription programs in RSK1 and RSK2 knockout cells using microarray analysis (Yang et al.). Their analysis demonstrated that RSK1 has specific roles in cell adhesion, cell cycle regulation and DNA replication and repair pathways. They further validated the functional significance of these identified transcriptional programs through cellular assays using mRNA datasets from cancer patients. Specifically, they discovered that RSK1 modulates the mRNA and protein expression of fibronectin1, affecting cell adhesion and CDK2, affecting S-phase arrest in the cell cycle, and impairing DNA replication and repair. In contrast, RSK2 showed increased ISG15 transcriptional expression, affecting the immune response and cytokine expression. In conclusion, RSK1 and RSK2-mediated isoform-specific transcriptional programs in cancers point to the imminent needs of isoform-specific inhibitors to target RSK functions more precisely with reduced side-effects.

Zhang L group examined the role of Mek/Erk/p90RSK pathway in Epstein-Brr virus (EBV) LMP1-C terminal binding affibody molecule-mediated growth suppression of nasopharyngeal carcinoma (NPC) using mouse tumor xenograft models (Guo et al.). Affibody molecules are a class of small (6.5 kDa) non-immunoglobulin affinity proteins generated by combination library of the three-helix scaffold of the Z domain derived from staphylococcal protein A. The small size gives these molecules an advantage of deep penetration and better pharmacokinetics in comparison to antibodies. The authors found that LMP1-C277 showed higher antitumor efficacy and dramatically suppressed the phosphorylation of Mek1/2, Erk1/2 and p90RSK, as well as the downstream transcription factor c-fos. These findings not only highlighted the important carcinogenic role of p90RSK but also provided evidence supporting the therapeutic role of LMP1-C277 in EBV-associated NPC.

Lee Y group evaluated the role of Erk/p90RSK in the regulation of capicua (CIC) during oncogenesis (Park et al.). CIC is a transcriptional repressor involved in the regulation of various developmental processes and the pathogenesis of diverse diseases including cancers. The authors found that Erk/p90RSK induced CIC cytoplasmic translocation and inactivated CIC activity. Intriguingly, p90RSK phosphorylated twelve serine (S) and threonine (T) residues within CIC, including S173 and S301, which in turn induced the cytoplasmic translocation of CIC. Replacing these 12 S/T residues with alanine abrogated the effects of p90RSK leading to enhanced tumor suppressing activity of CIC. These results further illuminate a pivotal role of p90RSK signaling in cancers.

Lin L group provided a comprehensive review regarding p90RSK-mediated inter- and intracellular signaling in kidney diseases (Lin and Hu). Chronic kidney disease (CKD), histologically characterized by fibrosis and inflammation, is one of the most common diseases in the world without specific treatment. CKD has a diverse etiology; however, its pathogenesis remains largely unknown. Both interstitial fibroblasts and tubular epithelial cells play essential roles in CDK pathogenesis and progression. Structurally, fibroblasts reside in the renal interstitium surrounding the tubules formed by epithelial cells. This proximity facilitates interstitial fibroblast-epithelial communication and interactions that are fundamental in maintaining the integrity of the kidney structure and environment, as well as fine-regulated process of adaptation to pathogenic cues. In a novel fibroblast-specific wildtype p90RSK-transgenic mouse model, p90RSK has been found to accelerate obstruction-induced renal fibrogenesis by inducing fibroblast-mediated epithelial apoptosis and transdifferentiation through reactive oxygen species (ROS) (Lin et al., 2019b; Shi et al., 2023) and forkhead box class O1 (FOXO1) pathway (Lin et al., 2019b). Specifically, p90RSK-overexpressing fibroblasts produce and release excessive H_2_O_2_, causing ROS accumulation and β-catenin nuclear translocation in the surrounding epithelial cells. Nuclear β-catenin not only interacts with transcription factor FOXO1 to promote tubular epithelial apoptosis but also triggers aberrant epithelial transdifferentiation or EMT through inducing TCF/LET-mediated gene expression, leading to kidney structural destruction and eventually fibrosis. These results illuminate a novel mechanism regarding the pivotal role of p90RSK-mediated fibroblast-epithelial communications in CKD development and progression. In addition to H_2_O_2_, extracellular vesicles (EVs) are also implicated in p90RSK-mediated intercellular communications since p90RSK-overexpressing fibroblasts-derived EVs were delivered into cocultured tubular epithelial cells. p90RSK also modulates multiple intracellular signaling events in mediating kidney fibrosis and inflammation: 1) phosphorylates and inactivates GSK-3β to induce fibroblast proliferation; 2) phosphorylates BAD to enhance fibroblast survival; and 3) phosphorylates p38 to increase the survival of M1 macrophages. Additionally, p90RSK signaling has also been involved in the pathogenesis of various kidney disease including diabetic nephropathy, glomerular diseases and other kidney diseases including hypocitraturia, kidney stone, virus-induced kidney injury, as well as renal cell carcinoma (RCC). These evidences support an essential role of p90RSK in mediating a complex intercellular and intracellular signaling network to modulate diverse cellular processes to initiate various progressive kidney diseases.

In summary, the selected articles in this Research Topic highlight the role and mechanism of p90RSK in diseases and provide helpful guidance for future research on these aspects and throw lights on future development of specific and efficient therapeutic strategies.
